# Genome-wide identification and analysis of wheat LRR-RLK family genes following Chinese wheat mosaic virus infection

**DOI:** 10.3389/fpls.2022.1109845

**Published:** 2023-01-17

**Authors:** Shuang Liu, Jiajia Lei, Juan Zhang, Hanhong Liu, Zhuangxin Ye, Jin Yang, Qiseng Lu, Peng Liu, Jianping Chen, Jian Yang

**Affiliations:** ^1^ College of Agriculture and Biotechnology, Zhejiang University, Hangzhou, China; ^2^ State Key Laboratory for Managing Biotic and Chemical Threats to the Quality and Safety of Agro-products, Institute of Plant Virology, Ningbo University, Ningbo, China; ^3^ Key Laboratory of Biotechnology in Plant Protection of MARA and Zhejiang Province, Institute of Plant Virology, Ningbo University, Ningbo, China; ^4^ Junan County Bureau of Agriculture and Country, Linyi, China

**Keywords:** LRR-RLKs, wheat, genome-wide, CWMV, resistance, plant hormone

## Abstract

**Background:**

As the largest plant receptor-like protein kinase (RLK) superfamily, the leucine-rich repeat receptor-like kinases (LRR-RLKs) family are involved in plant growth, development, and stress responses. However, the functions of LRR-RLKs in wheat immunity remain unknown.

**Results:**

In the current study, 929 LRR-RLKs were identified in *Triticum aestivum* genome database using the BLAST and hidden Markov models (HMM) approach and divided into 14 clades. Chromosomal localization and synteny analysis revealed that *TaLRR-RLKs* were randomly distributed on all chromosomes with 921 collinear events. Through the cis-acting elements analysis, we observed that *TaLRR-RLKs* participated in hormone response, light response, development, metabolism, and response to environmental stress. The transcript level of 14 random selected *TaLRR-RLKs* from each subfamily was regulated by plant hormone treatment and Chinese wheat mosaic virus (CWMV) infection. The function of *TaLRR-RLKs* in wheat resistance to CWMV infection was further investigated by virus-induced gene silencing assay. Additionally, the accumulation of MeJA response genes, as well as CWMV RNA were not changed in the *TaLRR-RLK* silencing plants under MeJA treatment.

**Conclusions:**

Our results demonstrated that TaLRR-RLKs play an important role in wheat resistance to viral infection *via* hormone signals and lay the groundwork for the functional study of TaLRR-RLKs in wheat.

## Introduction

During plant growth and development, a series of signal transduction events was triggered by the abundant members of the receptor-like kinase (RLK) superfamily (Shiu and Bleecker, 2001a; Gou et al., 2010). The RLK in higher plant was first found in maize (*Zea mays*) (Walker and Zhang, 1990) and subsequently identified in other plants, including *Arabidopsis* and rice (Haffani et al., 2004). At least 21 different families of RLK superfamily have been identified based on the amino acid sequences of N-terminal extracellular domains (ECD). Among them, the *LRR-RLK* family is the largest and most highly conserved family of RLKs in plant species (Shiu and Bleecker, 2001a). The structural features of the *LRR-RLK* family commonly comprise a typical leucine-rich repeat domains (LRR), a single-pass transmembrane domain (TM), and an intracellular protein kinase catalytic domain (PK) (Shiu and Bleecker, 2001b; Kobe and Kajava, 2001; Gou et al., 2010; Liu et al., 2017). LRRs consist of a universal structural motif of 20-30 amino acids that form tandem-repeat domains and contain at least eight different classes of LRR sequence in diverse numbers and permutations (Shiu and Bleecker, 2001a; Shiu and Bleecker, 2001b). Additionally, each LRR domain forms an α/β helix that locates on protein surface and participates in protein-protein interactions (Kobe and Deisenhofer, 1994; Shiu and Bleecker, 2001a). PK domain with approximately 250-300 amino acids contains two subdivisions, including protein-serine/threonine kinases and protein tyrosine kinases. PK domain is further divided into 12 sub-domains which participate in catalytic activity, specifically functions related to cell development (Hanks and Hunter, 1995).

Plant *LRR-RLKs* are currently predicted to be involved in two major functional categories. On the one hand, *LRR-RLKs* play crucial roles in plant-organ growth and development, including morphogenesis, organogenesis, and hormone signaling. For example, the establishment of cotyledon primordia is regulated by two members of *LRR-RLKs* in *Arabidopsis* (*RPK1* and *TOAD2*) (Nodine et al., 2007; Nodine and Tax, 2008). In addition, BRASSINOSTEROID INSENSITIVE (BRI1) and BRI1 ASSOCIATED RECEPTOR KINASE 1 (BAK1) are involved in brassinosteroid signal transduction (He et al., 2007). The LRR domain of receptor-like protein kinase 1 (RPK1) is involved in regulating abscisic acid (ABA) sensitivity (Osakabe et al., 2005). On the other hand, *LRR-RLKs* participate in biotic and abiotic stress response processes and therefore related to defense mechanisms (Wei et al., 2015). For instance, FLS2, a well-studied LRR-RLK, functions as a pattern-recognition receptor to perceive and bind bacterial flagellin (flg22), resulting in the signal transduction and amplification by the mitogen-activated protein kinase cascade to response the phosphorylation of intracellular kinases. WRKY22 and WRKY29 act downstream of FLS2 to activate defense-related genes (Asai et al., 2009). In addition, EFR, a cell-surface RLK of *Arabidopsis*, recognizes an epitope (elf18) of bacteria, activating plant defense responses (Zipfel et al., 2006; Schoonbeek et al., 2015). In wheat, the LRR-RLK gene *TaXa21*, which is related to *TaWRKY76* and *TaWRKY62*, plays a positive role in resistance to *Puccinia striiformis* f. sp. *Tritici* in high-temperature seedlings (Wang et al., 2019). Moreover, some *LRR-RLK* genes, such as *SOMATIC EMBRYOGENESIS RECEPTOR-LIKE KINASE* (*SERK*) and *ERECTA* (*ER*), possess dual functions in plant development and defense processes owing to the recognition of multiple ligands by one receptor (Godiard et al., 2003; Shpak et al., 2004; Colcombet et al., 2005; Afzal et al., 2008).

The Chinese wheat mosaic virus (CWMV), belonging to the genus *Furovirus*, family *Virgaviridae*, is an RNA virus containing two single-strand positive-sense genomic RNAs. CWMV RNA1 encodes three proteins: a replication-associated protein, an RNA-dependent RNA polymerase (RdRp), and a movement protein (MP). CWMV RNA2 encodes four proteins: a major capsid protein (CP), two minor CP-related proteins (N-CP and CP-RT), and a cysteine-rich protein (CRP) (Diao et al., 1999; Andika et al., 2013). Moreover, infectious full-length cDNA clones of CWMV have been constructed, which promote systemic CWMV infection in both wheat and *Nicotiana benthamiana* (Yang et al., 2016). In China, CWMV often mixed with wheat yellow mosaic virus to infect the host plants, causing significant symptoms and severe yield losses (Diao et al., 1999; Adams et al., 2009). However, the mechanisms of wheat resistance to CWMV infection are still largely unknown.

Recently, comprehensive analyses of the *LRR-RLKs* family have been performed in many plant species, including *Solanum lycopersicum* (Wei et al., 2015), *Arabidopsis* (Wu et al., 2016), soybean (Zhou et al., 2016), two citrus species (Magalhães et al., 2016), *Amborella trichopoda* (Liu et al., 2016), and five Rosaceae species (Sun et al., 2017). However, the phylogenetic and structural characteristics of the LRR-RLK superfamily in wheat have not yet been investigated. In the current study, we identified 929 putative wheat *LRR-RLK* genes in the wheat genome and comprehensively analyzed LRR-RLK phylogeny, motif conservation, protein structure, cis-acting elements, chromosomal loci and replication relationships using bioinformatics tools. The expression patterns of *TaLRR-RLK* genes in wheat responses to hormones and CWMV infection were also investigated. Moreover, we confirmed that the *TaLRR-RLKs* are involved in wheat resistance to CWMV infection. This study aims to provide a foundation for further functional investigations of *LRR-RLK*s in wheat defense response against viral infections.

## Materials and methods

### Genome-wide identification and characterization of *LRR-RLK* genes in wheat

To identify *LRR-RLK* genes in *T. aestivum*, the amino acid sequences of *Arabidopsis* (*AtLRR-RLKs*) and rice *LRR-RLKs* were used as queries to perform BLAST searches in Ensembl Plants database (http://plants.ensembl.org/) (Bolser et al., 2015). Additionally, we downloaded the corresponding hidden Markov models (HMM) of LRR and PK domains from the Pfam database version 32.0 (http://pfam.xfam.org/) (Finn et al., 2014) and used them as references to search for *LRR-RLKs* in the *T. aestivum* protein sequences from the International Wheat Genome Sequencing Consortium (release-55) (http://plants.ensembl.org/index.html). The results of these two approaches were combined to identify the *TaLRR-RLKs* wheat genome with an E-value < 10^-5^. All candidates were further screened *via* eliminating the redundant sequences and functional annotations and then analysis with the NCBI Batch Web CD-Search Tool (https://www.ncbi.nlm.nih.gov/Structure/bwrpsb/bwrpsb.cgi). The presence of signal peptides (SP) was calculated with SignalP (http://www.cbs.dtu.dk/services/SignalP/) (Petersen et al., 2011), the prediction of transmembrane (TM) domains was performed using the TMHMM server (http://www.cbs.dtu.dk/services/TMHMM/) (Krogh et al., 2001) and the subcellular localization of *TaLRR-RLKs* were predicted using ProtComp software on the SoftBerry online database (http://linux1.softberry.com/) (Long et al., 2014), respectively.

### Multiple sequence alignments and phylogenetic analysis of TaLRR-RLKs

Multiple sequence alignment of AtLRR-RLKs and TaLRR-RLKs was carried out using MEGA6 software (Tamura et al., 2013). Furthermore, an unrooted phylogenetic tree was generated with the maximum likelihood (ML) method by 1 000 bootstrap tests (Price et al., 2010). The TaLRR-RLKs were classified according to the topology of the phylogenetic trees and the established classification rely on *Arabidopsis* homologs (Shiu and Bleecker, 2001b). Pairwise deletion was used for data processing, while the Poisson distribution was adopted for tree-building model. The online tool iTol (http://itol.embl.de) (Letunic and Bork, 2011) was used to create improved graphical representations of the phylogenetic tree.

### Chromosomal localization and synteny analysis of *TaLRR-RLKs*

To investigate the distribution of *TaLRR-RLKs* in wheat chromosomes and gene duplication events, we downloaded wheat genomic sequences and genome annotation files from the Ensembl Plants database for further analysis. Subsequently, the chromosomal locations and synteny relationships of *TaLRR-RLKs* were illustrated using TBtools software using the Graphics function (Chen et al., 2020).

### Conserved-domain, motif, and protein tertiary structure analyses of TaLRR-RLKs

To better understand the protein features of each subfamily, all the identified TaLRR-RLKs were searched in Pfam database (Finn et al., 2014), and verified using CDD analysis (http://www.ncbi.nlm.nih.gov). To characterize the structural divergence of TaLRR-RLKs in each group, the conserved motifs in their encoded proteins were identified using TBtools software and the Simple Multiple Em for Motif Elicitation (MEME) Wrapper function (Chen et al., 2020). The maximum number of motifs was set to 10 and other optional parameters to default. The results of conserved domains and motif analysis of several representative proteins from each subfamily were displayed using TBtools (Chen et al., 2020), and then mapped in the clustered phylogenetic tree using Photoshop software. We used the automated SWISS-MODEL homology modeling server (https://swissmodel.expasy.org/) to generate three-dimensional protein models of the TaLRR-RLK proteins (Waterhouse et al., 2018). We randomly picked a gene from each group to conduct homology modeling based on QMEAN (-4 to 0, where the closer the value to 0, the better the match between the examined protein and the template) (Benkert et al., 2011) and GMQE (0 to 1, where the larger the value, the better the quality) (Waterhouse et al., 2018).

### Prediction and analysis of cis-acting elements of *TaLRR-RLKs*

The 2000 bp upstream of all *TaLRR-RLKs* coding sequences were identified from the Ensembl Plants database and then used predicted the conserved cis-acting elements in the putative promoter regions by the PlantCARE software (http://bioinformatics.psb.ugent.be/webtools/plantcare/html/) (Lescot et al., 2002). All identified cis-acting elements were clustered and arranged according to their different functions and visualized with the TBtools software (Chen et al., 2020).

### Plant materials and treatments

*T. aestivum*. cv. Yangmai 158 was grown in a glasshouse at 15 ± 2°C with a photoperiod of 16 h light, 8 h dark and 70% relative humidity (Yang et al., 2020). To analyze the expression profile of *TaLRR-RLKs* in wheat, we extracted total RNA from seven tissues (from bottom to top: root, stem, first leaf, second leaf, third leaf, fourth leaf, and fifth leaf) of five-leaf stage wheat seedlings for quantitative real-time PCR (qRT-PCR). To analyze the expression profile of *TaLRR-RLKs* under hormone treatments, wheat seedlings with two-leaf-stage were sprayed with 100 μmol L^–1^ abscisic acid (ABA), 100 μmol L^–1^ methyl jasmonate (MeJA), or distilled water (as control) (Yu et al., 2019). Three biological replicates of samples were collected at four different time after hormone treatment (2, 4, 6 and 12 h) for total RNA extraction. For investigate the expression level of *TaLRR-RLKs* in wheat leaves, the full-length cDNA clones of CWMV were used for CWMV inoculation according to a previous study (Yang et al., 2016). At 7 days post inoculation (dpi), the systemic leaves were sampled for RNA extraction and qRT-PCR assay.

### Virus-induced gene silencing in wheat

The VIGS tool function on the online website (https://solgenomics.net/)was used to obtain the best VIGS fragment sequence (300 bp) of *TaLRR-RLKs*. The specific fragments were amplified from wheat cDNA using RT-PCR (**Supplementary Table S1**), digested with *Pac*I and *Not*I restriction enzymes, and inserted into the BSMVγ gene (BSMV : TaLRR-RLK). Plasmids BSMV α and γ were linearized with *Mlu*I restriction enzyme, BSMV β with *Spe*I, and BSMV γ-gene with *BssH*II. Then, the linearized plasmid DNAs of BSMV RNAα, β, and γ were prepared for transcription *in vitro* using the T7 *in vitro* transcription kit (Ambion, Austin, TX; Promega, Shenzhen, China). Next, the transcription products were mixed at a 1:1:1 molar ratio, followed by addition of excess FES buffer (0.06 M potassium phosphate, 0.1 M glycine, 1% bentonite, 1% sodium pyrophosphate decahydrate, 1% celite, pH 8.5) for friction inoculation into second leaves of two-leaf-stage wheat plants, as previously described (Yang et al., 2020). Wheat seedlings inoculated with FES buffer were used as negative controls. Finally, the inoculated wheat was placed in a growth environment at 28 °C and 70% relative humidity for 7-10 days to observe the symptoms. Similarly, we mixed the transcripts of linearized plasmid DNAs of CWMV RNA R1 and R2 at a 1:1 molar ratio and inoculated third leaves of the BSMV-infected wheat plants. Subsequently, the virus inoculated wheat plants were grow for 21 days at 15 ± 2 °C and 70% relative humidity. At 10 dpi, viral infection leaves were harvested for RNA extraction. Photographs of viral infection leaves were taken at 40 dpi.

### Plant RNA isolation and qRT-PCR assay

Total RNA was extracted from each sample using a HiPure Plant RNA Mini Kit (Magen, Guangzhou, China). First Strand cDNA Synthesis Kit (Toyobo, Kita-ku, Osaka, Japan) with random primers was used to synthesize first-strand cDNA, and 1 µg total RNA was added per 20 µL reaction volume, as previously described (He et al., 2020). Hieff qPCR SYBR Green Master Mix (Yeasen, Shanghai, China) and ABI 7900HT sequence detection system (Applied Biosystems QuantStudio 5, Foster City, CA, USA) were used to perform quantitative PCR. The 2^-ΔΔCt^ method (Livak and Schmittgen, 2001) was used to quantify the relative gene expression. In addition, the expression of *T. aestivum* cell division cycle (*TaCDC*) gene (Accession Number: XM_020313450) was analyzed and used as an internal control (Zhang et al., 2019). Each treatment in this study had at least three biological and three technical replicates. All the gene-specific primers used in this study were listed in **Supplementary Table S1**.

### Statistical analysis

Microsoft Excel was used to determine the mean values and standard errors of the treatments. *t*-test or the Tukey’s test was performed using the SPSS 16.0 software (SPSS, Inc., Chicago, IL) to determine the significance of differences. The significant difference with unequal variance between two treatments was decided using the probability (P) value <0.05.

## Results

### Identification and classification of *LRR-RLK* genes in *T. aestivum*

**Figure 1:**
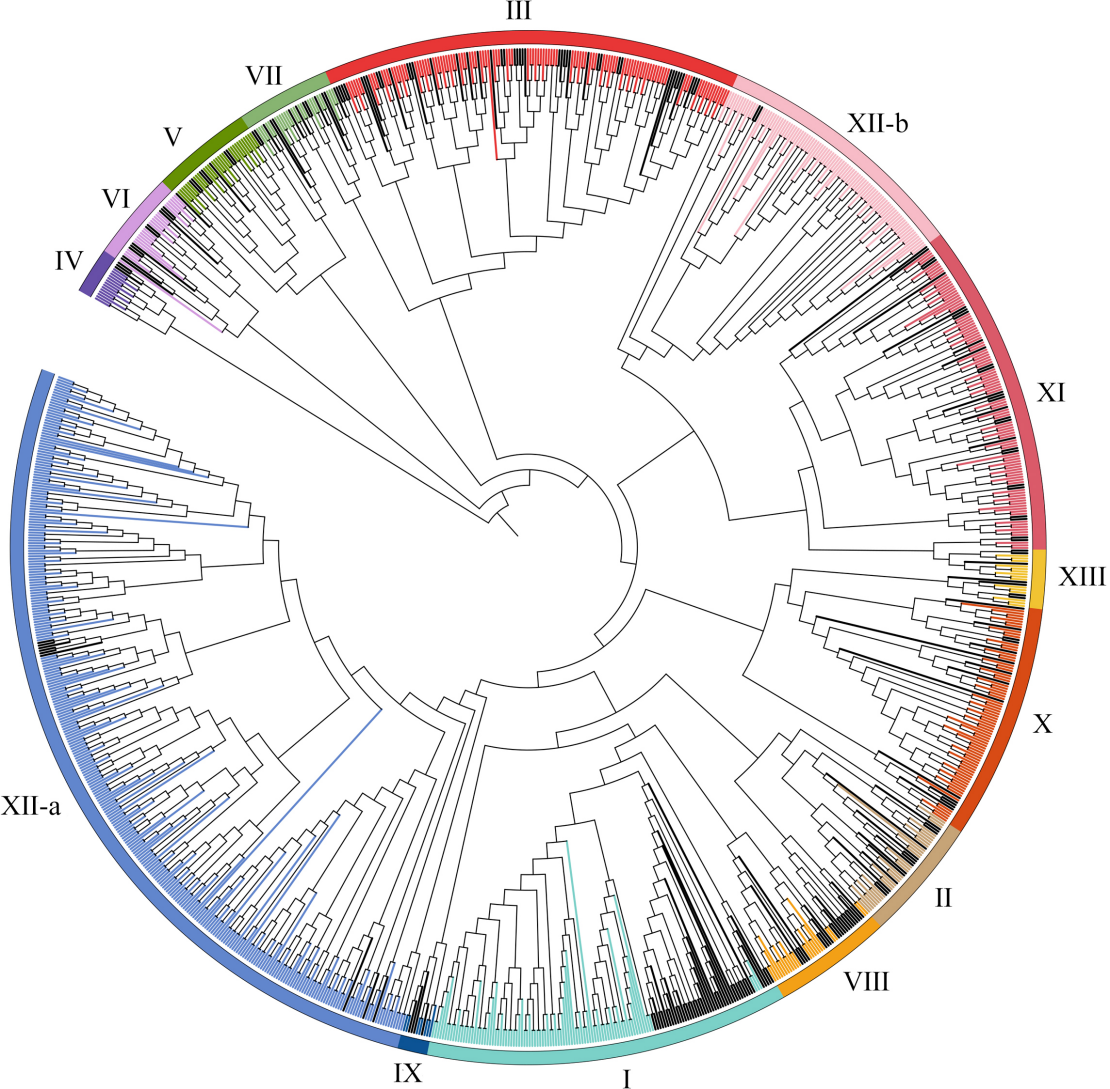
Phylogenetic tree for LRR-RLKs identified in *A. thaliana* and *T. aestivum*. The amino acid sequences of LRR-RLK from *A. thaliana* and *T. aestivum* were aligned by MEGA 6.0 and the phylogenetic tree was constructed using MEGA 6.0 by the maximum likelihood method with 1000 bootstrap replicates. A total of 929 LRR-RLK genes were classified into 14 groups (I-XI, XII-a, XII-b, XIII) and are highlighted by different colors. LRR-RLKs from *A. thaliana* in each subfamily were highlighted by black.

For purpose of identify the LRR-RLK family members in wheat, we used LRR-RLK sequences from the *A. thaliana* and rice genomes as a query to identify LRR-RLK sequences in the wheat genome database using the BLASTP and HMM approach. Total 6967 identified proteins were then submitted to the Pfam and SMART databases for analysis of domain structures. 929 candidates with at least two LRR domains and a kinase domain in the wheat genome were identified. The detailed information, including the accession number of each protein, were listed in **Supplementary Table S2**. To study the evolutionary relationships of TaLRR-RLKs, all 929 candidates and all AtLRR-RLK proteins was used to construct an ML tree (**Figure 1**). The results of the phylogenetic analysis illustrated that all TaLRR-RLKs were clustered in 14 major groups according to the nomenclature of the Arabidopsis homologues within the same group (I-XI, XII-a, XII-b, XIII) (**Figure 1**; **Supplementary Table S2**). Of these, subfamilies XII-a, III, XI, and XII-b were the top four largest subfamilies, with 305, 109, 96, and 93 members, respectively. The subfamilies IV and IX were the two smallest, with 14 and 7 members, respectively. Similar to phylogenetic tree in *Arabidopsis*, the members in LRR-XII fell into two different subfamilies (LRR-XIIa and LRR-XIIb). Although a few members belong to subfamily XII among AtLRR-RLKs, total 398 TaLRR-RLKs were identified as orthologs of these AtLRR-RLKs (**Figure 1**).

### Chromosomal locations and synteny analysis of *TaLRR-RLKs*

**Figure 2:**
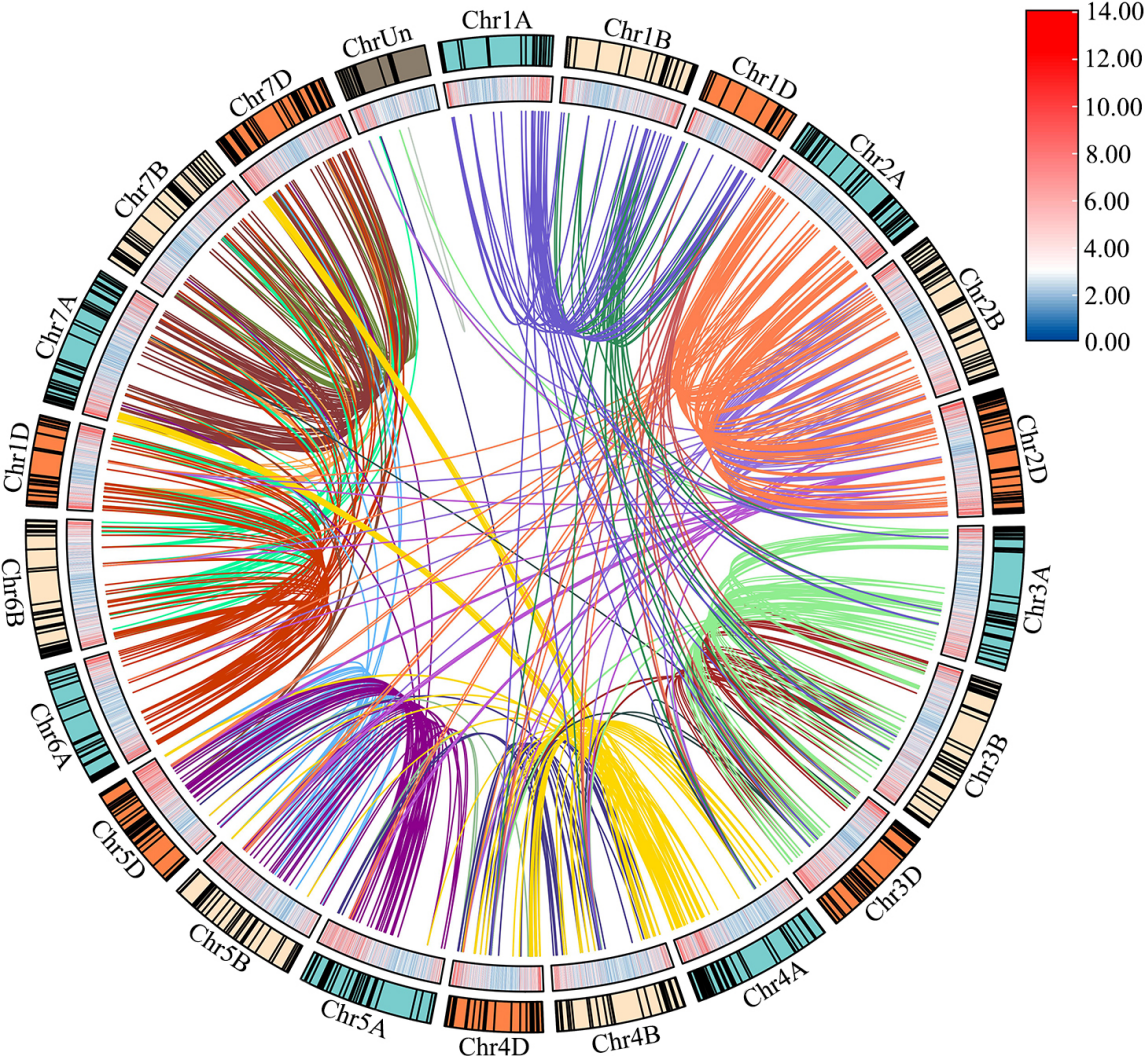
Chromosome distribution and duplication of the wheat LRR-*RLK* genes. Outer circle with colored blocks illustrates different chromosomes, and the short black lines indicate the location of these genes on chromosomes. The wheat gene density is displayed by heat map on the inner circle, and collinearity events are marked with different colored lines in center. The graphs of chromosomal location and synteny analysis were established using TBtools.

Based on the initial position of each gene on the wheat chromosomes, 929 *TaLRR-RLK* genes were randomly distributed among all 21 chromosomes (chromosomes 1A to 7D, Chr1A to Chr7D), with Chr2D having the most (65) and Chr4B having the least (30) genes (**Figure 2**; **Supplementary Table S3**). There were 16 *TaLRR-RLKs* identified on the unknown wheat chromosome (ChrUn). Combined with wheat gene density, the distribution of TaLRR-RLKs was not significantly correlated with the distribution of wheat genes. To explore duplication relationship of *TaLRR-RLKs* during evolution, we performed synteny analysis according to the position of each gene in wheat chromosomes. In our analysis, 123 genes were tandemly duplicated (**Supplementary Table S4**), and a total of 921 collinear events were identified and distinguished in different colors (**Figure 2**; **Supplementary Table S5**).

### Predicted structure analysis of TaLRR-RLK proteins

**Figure 3:**
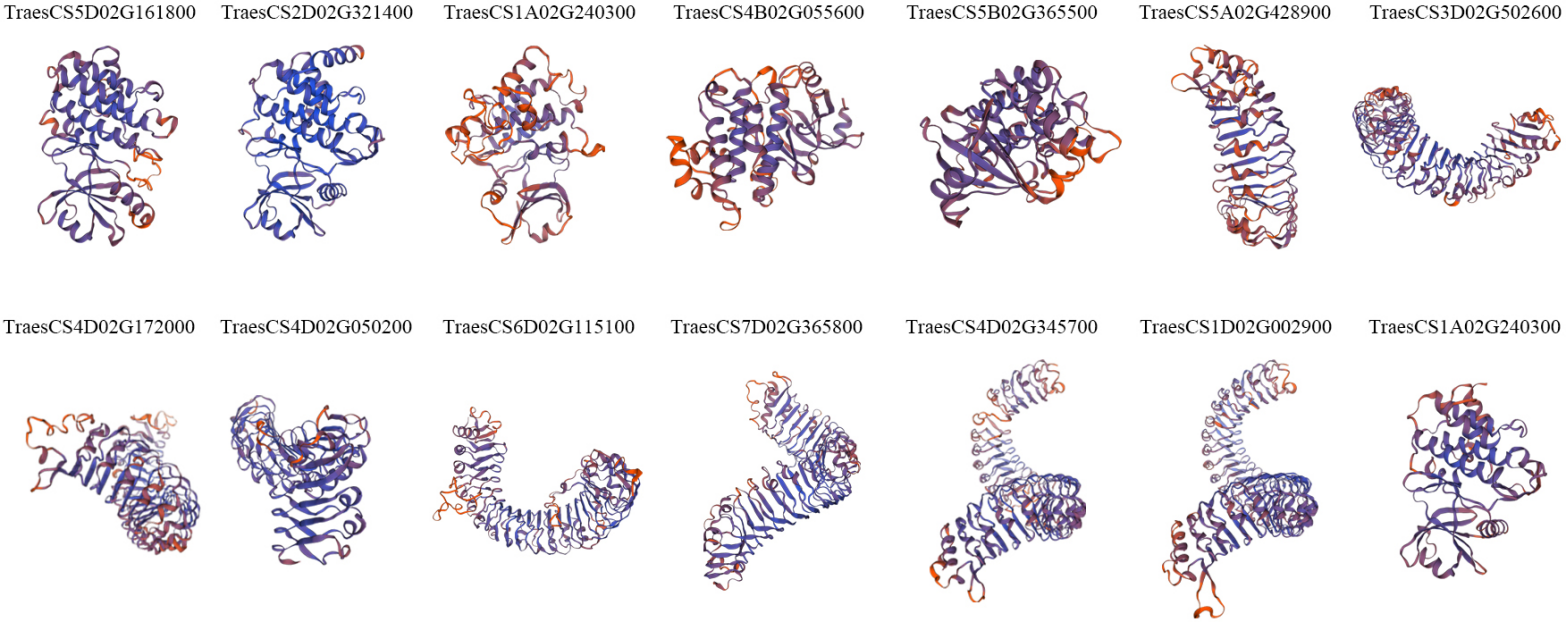
Prediction of the spatial structure of TaLRR-RLKs. The 14 TaLRR-RLKs was random selected from each subfamily. The SWISS-MODEL was used for structural prediction and the model with high confidence was selected based on QMEAN and GMQE.

To gain insight into the spatial conformations of TaLRR-RLKs, we used the SWISS-MODEL template library to conduct homology modeling and generate three-dimensional protein models. We randomly selected a TaLRR-RLK from each subfamily as a representative to analyze its predicted structure and illustrate the results in **Figure 3**. All 14 TaLRR-RLKs could be forecast as models, revealing that they maintained structural integrity during evolution, which is important for their function. Meanwhile, we found that the spatial structures of proteins from the same clade are highly similar.

### Protein domains and conserved motif analyses of wheat LRR-RLKs

**Figure 4:**
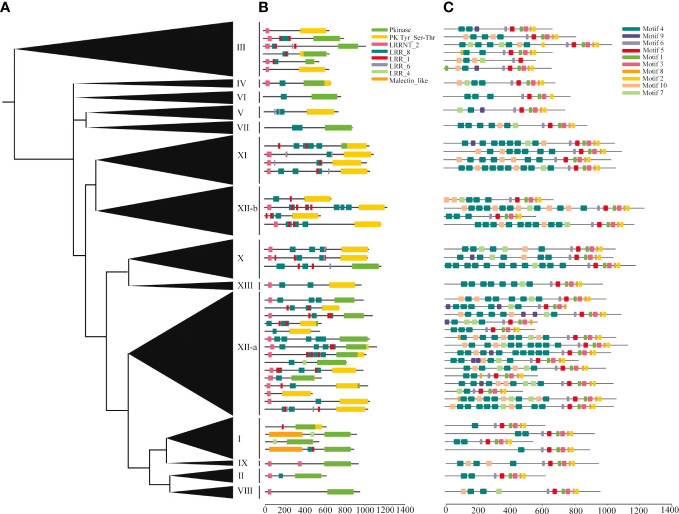
Protein domains and motif analyses of each LRR-RLK subgroup in *T. aestivum*. **(A)** Phylogenetic analysis of the TaLRR-RLKs using maximum likelihood. The phylogenetic tree with the subfamily names listed on the right. **(B)** Deduced structures of representative LRR-RLK proteins within each subfamily. LRR, Pkinase and malectin_like coding regions were marked by different colored rectangles. **(C)** MEME motif distribution of the representative proteins.

The deduced structures of representative proteins of each subfamily were mapped in the phylogenetic tree (**Figure 4A**), and the exact distributions of the LRR domains and PK domains identified for each TaLRR-RLK were listed in **Supplementary Tables S6**, **S7**, respectively. Our results showed that all members of each LRR-RLK subfamily contain multiple types of LRR domains. The frequency of LRR repeats was similar in each LRR-RLK subfamily and PK domains did not vary significantly across different subfamilies (**Figure 4B**, **Supplementary Tables S6**, **S7**). The most common types of LRR domain were LRR_8 (leucine-rich repeat 8), LRR_1 (leucine-rich repeat 1), and LRRNT_2 (leucine-rich repeat N-terminal 2), with different distribution patterns in each subfamily. For instance, subfamily IX preferentially contained LRRNT_2 but not LRR_1 and two LRR_8 domains were identified in this subfamily. Subfamily I preferentially contained LRR_8 and LRR_4 (leucine-rich repeat 4) but not LRRNT_2 or LRR_6 (leucine-rich repeat 6). Additionally, four subfamilies, including IV, VI, VIII, and IX did not contain LRR_4 or LRR_6. To further elucidate the structural characteristics and potential functions of LRR-RLKs in wheat, we set the threshold to 10 motifs in the MEME online tool to predict the putative motifs of these proteins. The distribution of these putative motifs in each representative TaLRR-RLK was shown in **Figure 4C**, and the detailed distribution was listed in **Supplementary Table S8**. Among them, motifs M6, M5, M1, M3, M8, and M2 were located in the PK domain. Motifs M1, M2, M3, M4, M5, and M6 were present in all members of each subfamily. The M4 motif was frequently detected in all TaLRR-RLK subfamilies, particularly in subfamilies XI, XII-a, and XII-b. The M9 motif had a dramatic presence in subfamilies I, II, IV, and VII. M1 appeared most frequently in subfamily XII-b. Meanwhile, we submitted protein sequences of each TaLRR-RLK subfamily to the SignalP website to search for possible signal peptides and found that 706 of the 929 TaLRR-RLKs contained SP, whereas 39% of the subfamily XII members did not contain signal peptides (**Supplementary Table S9**). Additionally, 865 genes contained at least one TM domain based on TMHMM prediction. Subcellular localization analysis showed that 900 TaLRR-RLKs were localized to the plasma membrane (**Supplementary Table S9**).

### Prediction and analysis of cis-acting elements of *TaLRR-RLKs*

To analysis of promoter elements of *TaLRR-RLKs*, we used PlantCARE to detected cis-acting regulatory elements of the promoter region (2000 bp sequence upstream of the translation start site) of the 929 LRR-RLKs. The results showed that a total of 80 putative cis-acting elements were prevalent in *TaLRR-RLKs*. The details of all genes was shown in **Table 1** and **Supplementary Table S10**. Briefly, the cis-acting elements predicted in the promoter regions were divided into seven categories (**Table 1**). The most abundant elements were phytohormone regulation-related cis-acting elements that respond to auxin, ABA, MeJA, gibberellins (GA) and salicylic acid (SA). The MeJA response element mainly included the CGTCA- and TGACG-motifs, and the ABA responsive element consisted of ABRE. Moreover, the TGA-element, TGA-box, AuXRR-core and AuxRE were involved in auxin responsiveness and the GARE-motif, P-box, and TATC-box were considered gibberellin-responsive elements involved in the regulation of gibberellin responsiveness. The second type of widely distributed regulatory elements were light responsive elements, including Box 4, G-Box, GATA-motif, TCT-motif, TCCC-motif, GT1-motif, and Sp1, of which G-Box appeared to be the most widespread. Moreover, we also identified cis-acting regulatory elements related to environmental stimuli, including abiotic and biotic stresses, of which LTR and TC-rich repeats were the most abundant (**Table 1**; **Supplementary Table S10**).

**Table 1:** Statistics analysis of cis-acting elements detected in promoter of *TaLRR-RLK* genes.

Types	Functional Classification	Element Species (ID of PlantCARE)	No. of Elements
1	Hormone responsive elements	ABRE, AuxRE, AuxRR-core, CGTCA-motif, GARE-motif, P-box, SARE, TATC-box, TCA-element, TGA-box, TGA-element, TGACG-motif	12
2	Light responsive elements	G-Box, TCT-motif, TCCC-motif, ACE, Box 4, Sp1, I-box, GT1-motif, chs-CMA1a, GATA-motif, LAMP-element, GA-motif, ATCT-motif, CAG-motif, chs-CMA2a, chs-CMA2c, Box II, C-box, chs-Unit 1 m1, ATC-motif, 3-AF1 binding site, GTGGC-motif, GATT-motif, Pc-CMA2c, AAAC-motif, chs-CMA2b, TGGCA, Gap-box, ACA-motif, Pc-CMA2a, L-box, AT1-motif, GGA-motif, LS7, sbp-CMA1c, 4cl-CMA2b, 4cl-CMA1c	38
3	Anaerobic elements	GC-motif, ARE	2
4	Development and metabolism-related elements	CAT-box, O2-site, GCN4_motif, NON-box, AACA_motif, RY-element, MSA-like, motif I, HD-Zip 1	9
5	Binding site elements	MBS, CCAAT-box, MRE,AT-rich element, MBSI, BOX III, HD-Zip 3	7
6	Environmental stress-related elements	TC-rich repeats, LTR, WUN-motif, DRE	4
7	Other elements	A-box, Box II -like sequence, CellCycle-1b, circadian, AT-rich sequence, 5UTR Py-rich stretch, 3-AF3 binding site, HMG-TATA-region	8

### Expression patterns of *TaLRR-RLKs* upon treatment with MeJA and ABA

**Figure 5:**
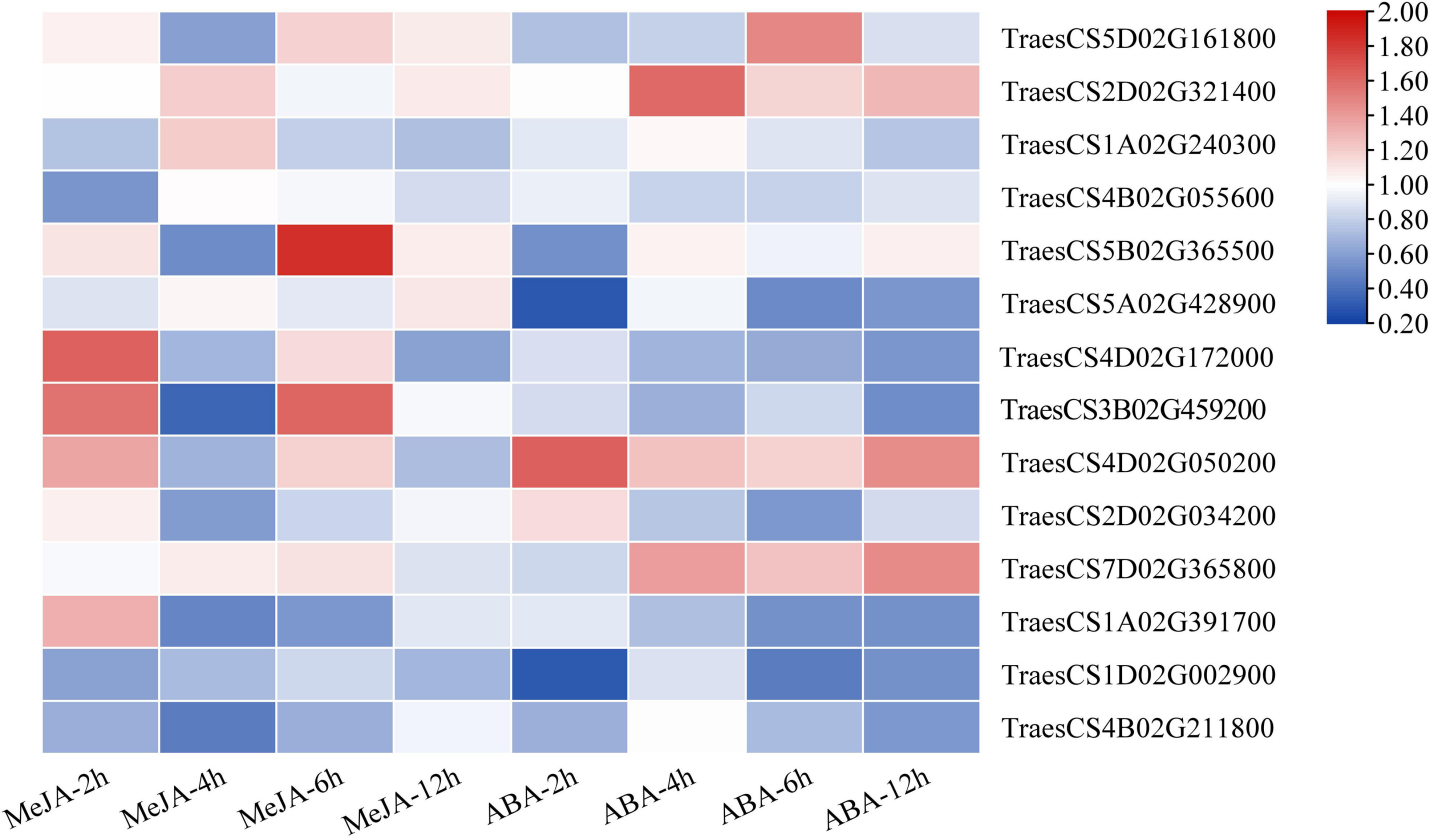
Relative expression analysis of 14 representative *TaRR-RLKs* from each subfamily in wheat leaves under MeJA and ABA treatment. Data shown are the means of three biological replicates ± the SE. The values were normalized to those for the reference gene (Cell Division Control protein, *TaCDC*) and are presented as fold changes in expression relative to that in wheat leaves without treatment. The mean expression values were visualized by Tbtools. The color scale represents expression values of each sample. The blue box represents the lower levels and the red box represent high levels of expression level. The qRT-PCR values used for the heat map are listed in **Supplementary Table S11**.

Based on the results of cis-acting regulatory element analysis, 12 types of hormone-responsive elements were identified (**Table 1**; **Supplementary Table S10**). Among them, the hormone-responsive elements related to MeJA and ABA were widely distributed in the promotor region of *TaLRR-RLKs* (**Supplementary Table S10**). To better understand the underlying functions of *TaLRR-RLK* response to MeJA and ABA, one *TaLRR-RLK* from each clade was randomly selected to analyze its respective expression level after hormone treatment using qRT-PCR. As shown in **Figure 5**, TraesCS5D02G161800(I), TraesCS1A02G240300(III), TraesCS4B02G055600(IV), TraesCS2D02G034200(X), and TraesCS7D02G365800(XI) were insensitive to MeJA and ABA. By contrast, the expression level of TraesCS5B02G365500(V) and TraesCS4D02G172000(VII) was significantly induced by MeJA, while that of TraesCS1A02G391700(XII-a) and TraesCS4B02G211800(XIII) was reduced. The expression levels of TraesCS3B02G459200(VIII) were significantly up-regulated at 2 hours post treatment (hpt) and 6 hpt, but down-regulated at 4 dpt by MeJA. Besides, the expression levels of TraesCS2D02G321400(II) and TraesCS4D02G050200(IX) were up-regulated after treatment with ABA, while TraesCS5A02G428900(VI) and TraesCS1D02G002900(XII-b) were down-regulated. The qRT-PCR values used for the heat map are listed in **Supplementary Table S11**. These results imply that *TaLRR-RLK* genes of *T. aestivum* participate in a variety of hormone responses (**Figure 5**).

### Tissue-specific analysis of *TaLRR-RLKs*

**Figure 6:**
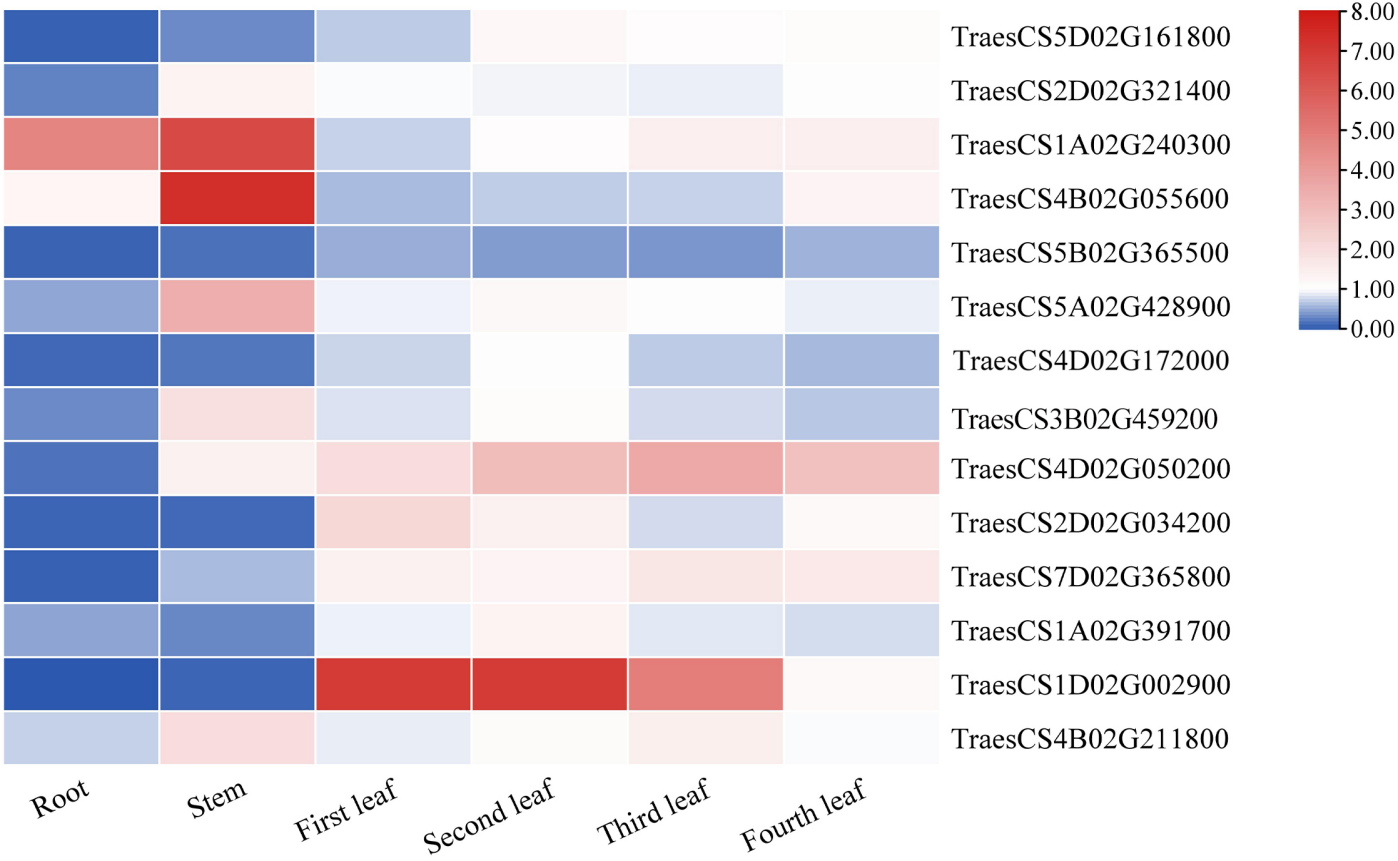
The differential expression of 14 representative *TaRR-RLKs* from each subfamily in different tissues was analyzed by qRT-PCR. The plants were divided into seven different tissues including root, stem, first leaf, second leaf, third leaf, fourth leaf, and fifth leaf and the fifth leaf as a control. Data shown are the means of three biological replicates ± the SE and visualized in TBtools. The values were normalized to those for *TaCDC* and are presented as fold changes in expression relative to that in wheat leaves without treatment. The mean expression values were visualized by Tbtools. The color scale represents expression values of each sample. The blue box represents the lower levels and the red box represent high levels of expression level. The qRT-PCR values used for the heat map are listed in **Supplementary Table S11**.

The plants were divided into seven different tissues: root, stem, first leaf, second leaf, third leaf, fourth leaf, and fifth leaf (bottom-top) to comprehensively explore the expression patterns of the *TaLRR-RLKs*. As shown in **Figure 6**, taking the fifth leaf as a control, all 14 random selected genes from each subfamily were differentially expressed in each tissue and most tended to be down-regulated in the roots. The expression level of TraesCS5B02G365500(V) was down-regulated in each tissue. In contrast, the expression level of TraesCS4D02G050200(IX) was high in all tissues except for the root. In addition to up-regulation in the stem, TraesCS4B02G211800(XIII) showed moderate expression levels in other tissues. The expression levels of TraesCS1A02G240300(III) showed to be significantly up-regulated in the root and stem. TraesCS1D02G002900(XII-b) was observed to be up-regulated in the first, second, and third leaves, whereas its expression level in the fourth leaf was down-regulated in the root and stem (**Figure 6**). The qRT-PCR values used for the heat map are listed in **Supplementary Table S11**.

### Functional analysis of *TaLRR-RLKs* in wheat resistance to CWMV infection

**Figure 7:**
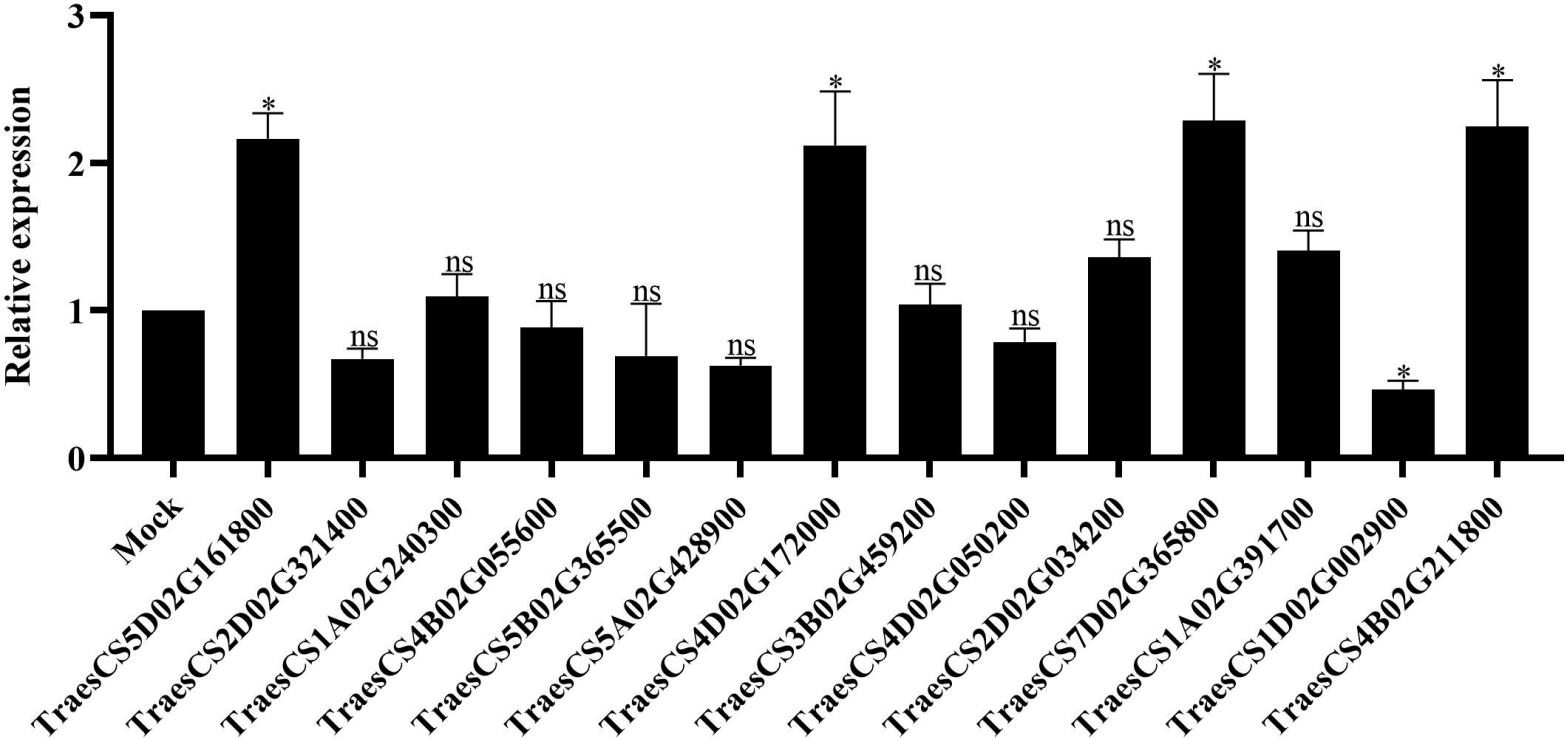
The transcript level of 14 representative TaLRR-*RLKs* in CWMV-inoculated wheat leaves. At 21 (days post inoculation), the viral infected leaves were sampled for qRT-PCR assay. Each result is the mean ± SE of three biological replicates. The transcript levels in the leaves without viral infection was used as mock and standardized as 1. The asterisks indicate significant difference were determined by student t-test (*P < 0.05). ns, no significant difference.

**Figure 8:**
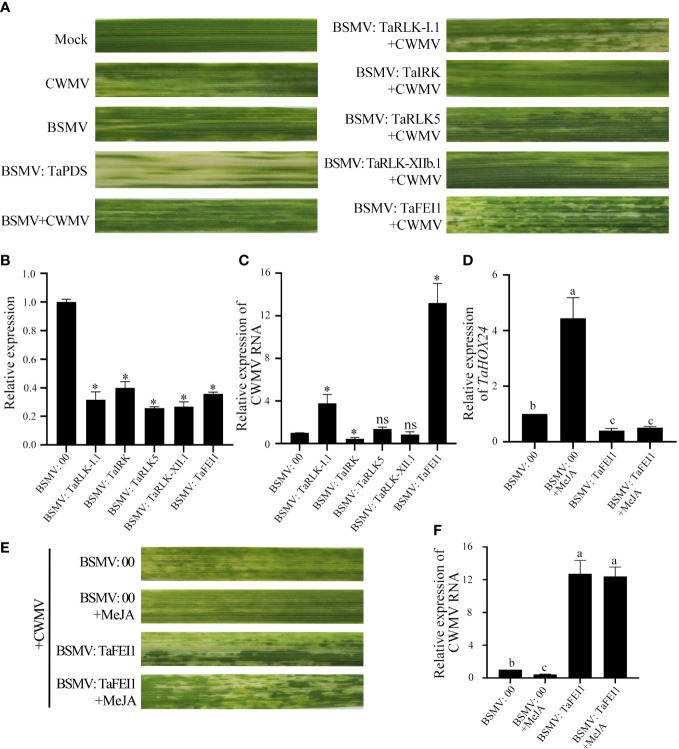
*TaLRR-RLKs* was involved in wheat resistance to CWMV infection. **(A)** Mild chlorotic mosaic symptoms were observed at 40 dpi on leaves inoculated with BSMV: 00, BSMV: TaPDS, BSMV: *TaRLK-I*1+CWMV, BSMV: *TaⅠRK*+CWMV, BSMV: *TaRLK5*+CWMV, BSMV: *TaRLK-XII*b.1+CWMV, BSMV: *TaFEI1*+CWMV, respectively. Mock, wheat leaves treated with 1×Fes buffer. Photographs were taken at 40 dpi. **(B)** Relative expression of *TaLRR-RLKs* during the interaction between *TaLRR-RLKs*-silencing plans and CWMV. **(C)** CWMV RNA accumulation in TaLRR-RLKs-silencing was analyzed by qRT-PCR using CWMV CP specific primers. **(D)** Relative expression of MeJA response gene TaHOX24 in TaFEI1-silencing plants under MeJA treatment. **(E)** Mild chlorotic mosaic symptoms were observed on leaves inoculated with BSMV: 00+CWMV or BSMV: *TaFEI1*+CWMV under MeJA treatment. Photographs were taken at 40 dpi. **(F)** The accumulation of CWMV RNA accumulation in TaFEI1-silencing plants under MeJA treatment was determined by qRT-PCR. For all qRT-PCR, the values were normalized to those for *TaCDC* and are presented as fold changes relative to that in BSMV: 00. Asterisks indicate significant differences between that in *TaLRR-RLKs*-silencing plants and mock using Student’s t-test or the Tukey’s test (P < 0.01). ns, no significant difference.

To investigate the function of *TaLRR-RLKs* in wheat resistance to CWMV infection, we analyzed the gene expression profile of *TaLRR-RLKs* under CWMV infection. The results indicated that four of selected 14 *TaLRR-RLKs* were differentially induced by CWMV infection, including TraesCS5D02G161800(I), TraesCS4D02G172000(VII), TraesCS7D02G365800(XI) and TraesCS4B02G211800(XIII). Besides, the accumulation level of TraesCS1D02G002900(XII-b) was significantly decreased under CWMV infection (**Figure 7**). Based on the homologous analysis of these five genes, we renamed TraesCS4D02G172000(VII) as *TaIRK*, TraesCS7D02G365800(XI) as *TaRLK5*, TraesCS4B02G211800(XIII) as *TaFEI1*, TraesCS5D02G161800(I) as *TaRLK-I.1* and TraesCS1D02G002900(XII-b) as *TaRLK-XIIb.1*, respectively. To further investigate the biological function of *TaRLKs* in CWMV infection, a BSMV-mediated gene silencing assay (BSMV-VIGS) was used to silence these five *TaLRR-RLKs*, respectively. We inoculated two-leaf-stage wheat seedlings with either BSMV:00+CWMV or BSMV : *TaLRR-RLKs*+CWMV. RT-PCR analysis verified the successful infection with BSMV and CWMV in all co-inoculated plants at 7 dpi (days post inoculation) (**Supplementary Figure S1**). After 40 dpi, the mosaic symptoms were observed in leaves of all virus infected wheat plants, and the mosaic symptoms that appeared on BSMV : TaFEI1+CWMV and TaRLK-I.1+CWMV co-inoculated plants were significantly stronger than those on BSMV:00+CWMV co-inoculated plants. BSMV : TaIRK+CWMV co-inoculated plants exhibited much less mosaic symptoms and BSMV : TaRLK5+CWMV and BSMV : TaRLK-XIIb.1+CWMV co-inoculated plants showed similar mosaic symptoms to BSMV:00+CWMV co-inoculated plants (**Figure 8A**). Moreover, qRT-PCR analysis with specific primers confirmed that the five *TaLRR-RLKs* were successfully silenced in all BSMV : TaLRR-RLKs+CWMV co-inoculated plants (**Figure 8B**). Furthermore, the accumulation of CWMV CP was significantly up-regulated in *TaFEI1*-silencing plants, but down-regulated in *TaIRK*-silencing plants (**Figure 8C**). These results indicate that *TaLRR-RLKs* play an important role in wheat resistance to CWMV infection. Because CWMV RNA accumulation was significantly induced in *TaFEI1-*silencing plants compared to that in other selected LRR-RLKs silencing plants, indicating that TaFEI1 is more important for wheat resistance against to CWMV infection (**Figures 8A**, **C**). TaFEI1 was selected for further study.

Considering that the expression levels of *TaFEI1* were significantly down-regulated after MeJA treatment, but not after ABA treatment (**Figure 5**; **Supplementary Table S11**), we hypothesized that the function of *TaFEI1* in CWMV infection were related to MeJA pathways. Thus, we calculated the accumulation of MeJA-response gene *TaHOX24* and CWMV RNA in *TaFEI1*-silencing plants after MeJA hormones treatment using CWMV CP specific primers. The results of qRT-PCR showed that *TaHOX24* was significantly induced in BSMV: 00 inoculated wheat leaves, but reduced in *TaFEI1*-silencing plants. However, MeJA treatment could not alter the relative expression level of *TaHOX24* in *TaFEI1*-silencing plants (**Figure 8D**). *TaFEI1*-silencing plants was then inoculated with CWMV together with MeJA treatment. Compared to wheat BSMV inoculated plants without MeJA treatment, CWMV RNA was significantly reduced by MeJA in BSMV: 00-inoculated plants, but not changed under MeJA treatment in BSMV: TaFEI1-inoculated plants (**Figure 8E**). At 40 dpi, BSMV: 00+CWMV co-inoculated wheat leaves under MeJA exhibited much less symptoms of mosaic disease compared to that without MeJA. However, MeJA treatment was not altered the symptoms of mosaic disease *TaFEI1*-silencing plants (**Figure 8F**). These results suggested that TaFEI1 may be involved in wheat resistance to CWMV infection dependent on MeJA signal pathway.

## Discussion

Identification of the *LRR-RLK* gene family at the genomic level using bioinformatics tools has contributed to the understanding of the function of *LRR-RLKs* in plant growth regulatory networks. In recent years, many studies have reported genome-wide identification and functional analysis of the *LRR-RLK* gene family in plant species, including *Saccharum* (Cheng et al., 2021), cotton (Sun et al., 2018), soybean (Zhou et al., 2016), and other species (Magalhães et al., 2016; Liu et al., 2016; Sun et al., 2017). In this study, 929 *TaLRR-RLKs* members were systematically identified in the *T. aestivum* genome and categorized into 14 clades reference to *AtLRR-RLKs* (**Figure 1**), suggesting that the wheat *LRR-RLK* genes may have similar functions with the *AtLRR-RLK* genes in the same clade. Additionally, compared with the *LRR-RLK* genes identified in *Arabidopsis*, wheat genome harbored more *LRR-RLK* genes of XII group (~42.84% in *TaLRR-RLKs*, while only ~4.05% in *AtLRR-RLKs*) and fewer genes of the VIII group (~2.48% in *TaLRR-RLKs*, while ~9.46% in *AtLRR-RLKs*) (**Supplementary Table S2**). These results suggested that sequence variation and biological function were significantly different in the *TaLRR-RLK* gene family. Similar to phylogenetic tree in *Arabidopsis*, the members in LRR-XII fell into two different subfamilies and could be rearranged to facilitate future functional analysis of their gene members (Gou et al., 2010). Moreover, phylogenetic analysis was help to evaluate functional redundancy of genes in one subfamily. For instance, SERK genes in subfamily LRR-XII has redundant function in male sporogenesis, pathogen response and cell death (Albrecht et al., 2008). And previous studies have reported that FEI1 and FEI2 in subfamily XIII construct signaling pathways that regulate cell wall function (Xu et al., 2008). Moreover, the results of motif prediction indicated that the types and the numbers of TaLRR-RLK protein motifs showed similarly distribution in a subfamily suggest a high degree of functional redundancy among TaLRR-RLK proteins in the same group (**Supplementary Table S8**). Thus, our results might guide researchers to overcome functional redundancy of genes in one subfamily when analyzed the function of *TaLRR-RLKs*. Additionally, we predicted the signal peptide, transmembrane domain and subcellular localization of each TaLRR-RLKs (**Supplementary Table S9**). These results provide a reference for further studies on the biological functions of *TaLRR-RLKs*.

The results of the synteny analysis showed that 921 collinear events were identified in *TaLRR-RLKs* (**Figure 2**; **Supplementary Table S5**), which may have contributed to the expansion of the *TaLRR-RLK* gene family. Of note, gene duplication events are vital to the expansion of gene families and the evolution or rearrangement of genomes, mainly owing to tandem, segment, and transposition duplications, which help organisms develop new biological functions and adapt to various environments (Zhang, 2003; Cannon et al., 2004; Moore and Purugganan, 2005). Here, we identified 123 tandem duplication events (**Supplementary Table S4**). The results revealed that tandem and segmental duplications contributed significantly to the expansion of the *TaLRR-RLK* gene family, whereby segmental duplication appears to be the essential duplication pattern. According to previous studies, cis-acting regulatory elements are vital molecular switches that participate in the transcriptional regulation of gene activities under phytohormones, various environmental factors, and photoreactions (Zan et al., 2013). The results of our cis-acting element analysis showed that the types of cis-acting regulatory elements were different for each *TaLRR-RLK* (**Table 1**; **Supplementary Table S10**). Accordingly, we suggested that *TaLRR-RLKs* may be widely involved in various tress response in wheat. Our results also showed that the promoter of *TaLRR-RLKs* also contained many cis-regulatory elements related to development and metabolism, such as CAT-box, O2-site, GCN4-motif, NON-box, AACA-motif, and RY element. Among these, AACA-motif is an enhancer element necessary for the specific expression of the glutelin gene in rice endosperm (Wu et al., 2000). In addition, all random selected *TaLRR-RLKs* were differentially expressed in all wheat tissues and tended to be down-regulated in the roots (**Figure 6**). It has been showed that LRR-RLKs play important roles in plant growth and development (Dievart et al., 2020). Thus, our results suggested that *TaLRR-RLKs* participated in wheat development and metabolism.

A number of *LRR-RLKs* were found to regulate plant innate immunity (Dievart et al., 2020). For instance, FLS2, EFR, PEPR1 and PEPR2 play key roles in defense responses as receptors for flagellin, EF-Tu, and endogenous Pep peptides, respectively (Gómez-Gómez and Boller, 2000; Zipfel et al., 2006; Yamaguchi et al., 2006; Yamaguchi et al., 2010; Krol et al., 2010). MOL1, a member of AtLRR-RLKs, is important in the homeostasis of *Arabidopsis* cambium *via* repressing the stress-related ethylene and jasmonic acid signaling pathways (Gursanscky et al., 2016). The SERK also plays a role in steroidal hormones BR signal transduction (He et al., 2007). In this study, silencing of *TaFEI1* and *TaRLK-I.1* significantly reduced the resistance of wheat resistance to CWMV infection, and the CWMV RNA accumulation was significantly reduced in wheat leaves of *TaIRK*-silencing plants. These results suggested *TaLRR-RLKs* play an important role in wheat antiviral response (**Figure 8**). Moreover, *LRR-RLKs* were involved in multiple signaling pathway regulation. For example, BAK1 not only functions as a co-receptor of BRI1, which is the BR receptor, but also participats in innate immune process *via* interacting with FLS2, EFR, PEPR1, and PEPR2 (Chinchilla et al., 2007; Heese et al., 2007; Li, 2010; Postel et al., 2010; Roux et al., 2011). Our results found the promoter region of *TaLRR-RLK* contained abundant MeJA and ABA hormone-responsive cis-acting elements (**Supplementary Table S10**). Either ABA or MeJA altered the relative expression level of several random selected *TaLRR-RLKs*, indicating that *TaLRR-RLKs* were involved in multiple plant hormone signals. Previous study showed that up-regulation of the JA pathway was one way of plant antiviral response (He et al., 2017). For example, In tobacco, silencing the JA biosynthesis gene AOS (ALLENE OXIDE SYNTHASE) enhanced plant resistance, and exogenous application of methyl jasmonate (MeJA) reduced resistance to TMV and allowed systemic movement (Oka et al., 2013). Moreover, JA-responsive genes are regulated after infection of CaMV and *Panicum* mosaic virus and its satellite virus at the early stages (2012; Love et al., 2005; Mandadi and Scholthof, 2012). In our study, the accumulation of CWMV RNA were not changed in the *TaFEI1*-silencing plants under MeJA treatment (**Figure 8**). Thus, we suggested that *TaLRR-RLKs* was play an important role in wheat resistance to viral infection in a hormone-dependent manner.

As one of the most important pathogenic agents causing wheat mosaic disease in China, CWMV normally caused 10-30% of yield losses, sometimes, up to 70% in severe cases (Guo et al., 2019). Up to now, the best countermeasure to control this disease is cultivation of resistant wheat varieties. However, the resistance genes to CWMV infection in wheat were still un-identified. To our knowledge, this is the first report that *TaLRR-RLKs* participate in wheat resistance to CWMV infection depend on plant hormone signals. This study provides insights into the molecular mechanisms of interaction between wheat and CWMV and offered a direction for identification of resistance genes in wheat response to CWMV infection.

## Data availability statement

The original contributions presented in the study are included in the article/**Supplementary Material**. Further inquiries can be directed to the corresponding authors.

## Author contributions

PL, SL, JiaY and JC conceived and designed the experiments. SL, JL, JZ, HL and ZY performed the experiments and collected the data. SL, JinY and QL analyzed the data and wrote results. SL and PL wrote the manuscript. All authors contributed to the article and approved the submitted version

## Supplementary material

Supplementary Figure 1 Detection of CWMV and BSMV by RT-PCR in viuses infected leaves. The infectious clones and wheat leaves without viral infection was used as positive control (+) and negative control (-), respectively.

## References

[B1] Adams M. J. Antoniw J. F. Kreuze J. (2009). Virgaviridae: a new family of rod-shaped plant viruses. Arch. Virol. 154, 1967–1972. doi: 10.1007/s00705-009-0506-6 19862474

[B2] Afzal A. J. Wood A. J. Lightfoot D. A. (2008). Plant receptor-like serine threonine kinases: Roles in signaling and plant defense. Mol. Plant Microbe Interact. 21, 507–517. doi: 10.1094/MPMI-21-5-0507 18393610

[B3] Albrecht C. Russinova E. Kemmerling B. Kwaaitaal M. De Vries S. C. (2008). *Arabidopsis* SOMATIC EMBRYOGENESIS RECEPTOR KINASE proteins serve brassinosteroid-dependent and -independent signaling pathways. Plant Physiol. 148, 611–619. doi: 10.1104/pp.108.123216 18667726 PMC2528080

[B4] Andika I. B. Sun L. Xiang R. Li J. Chen J. (2013). Root-specific role for *Nicotiana benthamiana* RDR6 in the inhibition of Chinese wheat mosaic virus accumulation at higher temperatures. Mol. Plant Microbe Interact. 26, 1165–1175. doi: 10.1094/MPMI-05-13-0137-R 23777430

[B5] Asai T. Tena G. Plotnikova J. Willmann M. R. Chiu W. L. Gomez-Gomez L.. (2009). MAP kinase signalling cascade in *Arabidopsis* innate immunity. Nature 415, 977–983. doi: 10.1038/415977a 11875555

[B6] Benkert P. Biasini M. Schwede T. (2011). Toward the estimation of the absolute quality of individual protein structure models. Bioinformatics 27, 343–350. doi: 10.1093/bioinformatics/btq662 21134891 PMC3031035

[B7] Bolser D. M. Kerhornou A. Walts B. Kersey P. (2015). Triticeae resources in ensembl plants. Plant Cell Physiol. 56, e3. doi: 10.1093/pcp/pcu183 25432969 PMC4301745

[B8] Cannon S. B. Mitra A. Baumgarten A. Young N. D. May G. (2004). The roles of segmental and tandem gene duplication in the evolution of large gene families in *Arabidopsis thaliana* . BMC Plant Biol. 4, 10. doi: 10.1186/1471-2229-4-10 15171794 PMC446195

[B9] Chen C. Chen H. Zhang Y. Thomas H. R. Frank M. H. He Y.. (2020). TBtools: an integrative toolkit developed for interactive analyses of big biological data. Mol. Plant 13, 1194–1202. doi: 10.1016/j.molp.2020.06.009 32585190

[B10] Cheng W. Wang Z. Xu F. Ahmad W. Lu G. Su Y.. (2021). Genome-wide identification of LRR-RLK family in saccharum and expression analysis in response to biotic and abiotic stress. Curr. Issues Mol. Biol. 43, 1632–1651. doi: 10.3390/cimb43030116 34698114 PMC8929030

[B11] Chinchilla D. Zipfel C. Robatzek S. Kemmerling B. Nürnberger T. Jones J. D.. (2007). A flagellin-induced complex of the receptor FLS2 and BAK1 initiates plant defence. Nature 448, 497–500. doi: 10.1038/nature05999 17625569

[B12] Colcombet J. Lelievre F. Thomine S. Barbier-Brygoo H. Frachisse J. M. (2005). Distinct pH regulation of slow and rapid anion channels at the plasma membrane of *Arabidopsis thaliana* hypocotyl cells. J. Exp. Bot. 56, 1897–1903. doi: 10.1093/jxb/eri184 15928017

[B13] Diao A. P. Chen J. P. Ye R. Zheng T. Yu S. Q. Antoniw J. F.. (1999). Complete sequence and genome properties of Chinese wheat mosaic virus, a new furovirus from China. J. Gen. Virol. 80, 1141–1145. doi: 10.1099/0022-1317-80-5-1141 10355760

[B14] Dievart A. Gottin C. Perin C. Ranwez V. Chantret N. (2020). Origin and diversity of plant receptor-like kinases. Annu. Rev. Plant Biol. 71, 131–156. doi: 10.1146/annurev-arplant-073019-025927 32186895

[B15] Finn R. D. Bateman A. Clements J. Coggill P. Eberhardt R. Y. Eddy S. R.. (2014). Pfam: the protein families database. Nucleic Acids Res. 42, D222–D230. doi: 10.1093/nar/gkt1223 24288371 PMC3965110

[B16] Godiard L. Sauviac L. Torii K. U. Grenon O. Mangin B. Grimsley N. H.. (2003). ERECTA, an LRR receptor-like kinase protein controlling development pleiotropically affects resistance to bacterial wilt. Plant J. 36, 353–365. doi: 10.1046/j.1365-313X.2003.01877.x 14617092

[B17] Gómez-Gómez L. Boller T. (2000). FLS2: an LRR receptor-like kinase involved in the perception of the bacterial elicitor flagellin in *Arabidopsis* . Mol. Cell. 5, 1003–1011. doi: 10.1016/S1097-2765(00)80265-8 10911994

[B18] Gou X. He K. Yang H. Yuan T. Lin H. Clouse S. D.. (2010). Genome-wide cloning and sequence analysis of leucine-rich repeat receptor-like protein kinase genes in *Arabidopsis thaliana* . BMC Genomics 11, 19. doi: 10.1186/1471-2164-11-19 20064227 PMC2817689

[B19] Guo L. M. He J. Li J. Chen J. P. Zhang H. M. (2019). Chinese Wheat mosaic virus: A long-term threat to wheat in China. J. Integr. Agr. 18, 821–829. doi: 10.1016/S2095-3119(18)62047-7

[B20] Gursanscky N. R. Jouannet V. Grünwald K. Sanchez P. Laaber-Schwarz M. Greb T.. (2016). MOL1 is required for cambium homeostasis in. Arabidopsis. Plant J. 86, 210–220. doi: 10.1111/tpj.13169 26991973 PMC5021142

[B21] Haffani Y. Z. Silva N. F. Goring D. R. (2004). Receptor kinase signalling in plants. Botany 82, 1–15. doi: 10.1139/b03-126

[B22] Hanks S. K. Hunter T. (1995). The eukaryotic protein kinase superfamily: kinase (catalytic) domain structure and classification. FASEB J. 9, 576–596. doi: 10.1096/fasebj.9.8.7768349 7768349

[B23] He L. Chen X. Yang J. Zhang T. Li J. Zhang S.. (2020). Rice black-streaked dwarf virus-encoded P5-1 regulates the ubiquitination activity of SCF E3 ligases and inhibits jasmonate signaling to benefit its infection in rice. New Phytol. 225, 896–912. doi: 10.1111/nph.16066 31318448 PMC6972624

[B24] Heese A. Hann D. R. Gimenez-Ibanez S. Jones A. M. He K. Li J.. (2007). The receptor-like kinase SERK3/BAK1 is a central regulator of innate immunity in plants. Proc. Natl. Acad. Sci. U. S. A. 104, 12217–12222. doi: 10.1073/pnas.0705306104 17626179 PMC1924592

[B25] He K. Gou X. Yuan T. Lin H. Asami T. Yoshida S.. (2007). BAK1 and BKK1 regulate brassinosteroid-dependent growth and brassinosteroid-independent cell-death pathways. Curr. Biol. 17, 1109–1115. doi: 10.1016/j.cub.2007.05.036 17600708

[B26] He Y. Zhang H. Sun Z. Li J. Hong G. Zhu Q.. (2017). Jasmonic acid-mediated defense suppresses brassinosteroid-mediated susceptibility to rice black streaked dwarf virus infection in rice. New Phytol. 214, 388–399. doi: 10.1111/nph.14376 27976810

[B27] Kobe B. Deisenhofer J. (1994). The leucine-rich repeat: a versatile binding motif. Trends Biochem. Sci. 19, 415–421. doi: 10.1016/0968-0004(94)90090-6 7817399

[B28] Kobe B. Kajava A. V. (2001). The leucine-rich repeat as a protein recognition motif. Curr. Opin. Struct. Biol. 11, 725–732. doi: 10.1016/S0959-440X(01)00266-4 11751054

[B29] Krogh A. Larsson B. Von Heijne G. Sonnhammer E. L. (2001). Predicting transmembrane protein topology with a hidden Markov model: Application to complete genomes. J. Mol. Biol. 305, 567–580. doi: 10.1006/jmbi.2000.4315 11152613

[B30] Krol J. Loedige I. Filipowicz W. (2010). The widespread regulation of microRNA biogenesis, function and decay. Nat. Rev. Genet. 11, 597–610. doi: 10.1038/nrg2843 20661255

[B31] Lescot M. Dehais P. Thijs G. Marchal K. Moreau Y. Van De Peer Y.. (2002). PlantCARE, a database of plant cis-acting regulatory elements and a portal to tools for in silico analysis of promoter sequences. Nucleic Acids Res. 30, 325–327. doi: 10.1093/nar/30.1.325 11752327 PMC99092

[B32] Letunic I. Bork P. (2011). Interactive tree of life v2: online annotation and display of phylogenetic trees made easy. Nucleic Acids Res. 39, W475–W478. doi: 10.1093/nar/gkr201 21470960 PMC3125724

[B33] Li J. (2010). Multi-tasking of somatic embryogenesis receptor-like protein kinases. Curr. Opin. Plant Biol. 13, 509–514. doi: 10.1016/j.pbi.2010.09.004 20926334

[B34] Liu P. L. Du L. Huang Y. Gao S. M. Yu M. (2017). Origin and diversification of leucine-rich repeat receptor-like protein kinase (LRR-RLK) genes in plants. BMC Evol. Biol. 17, 47. doi: 10.1186/s12862-017-0891-5 28173747 PMC5296948

[B35] Liu P. L. Xie L. L. Li P. W. Mao J. F. Liu H. Gao S. M.. (2016). Duplication and divergence of leucine-rich repeat receptor-like protein kinase (LRR-RLK) genes in basal *Angiosperm amborella trichopoda* . Front. Plant Sci. 7, 1952. doi: 10.3389/fpls.2016.01952 28066499 PMC5179525

[B36] Livak K. J. Schmittgen T. D. (2001). Analysis of relative gene expression data using real-time quantitative PCR and the 2^–ΔΔCT^ method. Methods 25, 402–408. doi: 10.1006/meth.2001.1262 11846609

[B37] Long R. Wang H. Shen Y. Kang J. Zhang T. Sun Y.. (2014). Molecular cloning and functional analysis of a salt-induced gene encoding an RNA-binding protein in alfalfa. Mol. Breed. 34, 1465–1473. doi: 10.1007/s11032-014-0130-3

[B38] Love A. J. Geri C. Laird J. Carr C. Yun B. W. Loake G. J.. (2012). Cauliflower mosaic virus protein P6 inhibits signaling responses to salicylic acid and regulates innate immunity. PloS One 7, e47535. doi: 10.1371/journal.pone.0047535 23071821 PMC3469532

[B39] Love A. J. Yun B. W. Laval V. Loake G. J. Milner J. J. (2005). Cauliflower mosaic virus, a compatible pathogen of arabidopsis, engages three distinct defense-signaling pathways and activates rapid systemic generation of reactive oxygen species. Plant Physiol. 139, 935–948. doi: 10.1104/pp.105.066803 16169957 PMC1256007

[B40] Magalhães D. M. Scholte L. L. S. Silva N. V. Oliveira G. C. Zipfel C. Takita M. A.. (2016). LRR-RLK family from two citrus species: genome-wide identification and evolutionary aspects. BMC Genomics 17, 623. doi: 10.1186/s12864-016-2930-9 27515968 PMC4982124

[B41] Mandadi K. K. Scholthof K. B. (2012). Characterization of a viral synergism in the monocot *Brachypodium distachyon* reveals distinctly altered host molecular processes associated with disease. Plant Physiol. 160, 1432–1452. doi: 10.1104/pp.112.204362 22961132 PMC3490591

[B42] Moore R. C. Purugganan M. D. (2005). The evolutionary dynamics of plant duplicate genes. Curr. Opin. Plant Biol. 8, 122–128. doi: 10.1016/j.pbi.2004.12.001 15752990

[B43] Nodine M. D. Tax F. E. (2008). Two receptor-like kinases required together for the establishment of *Arabidopsis* cotyledon primordia. Dev. Biol. 314, 161–170. doi: 10.1016/j.ydbio.2007.11.021 18158146

[B44] Nodine M. D. Yadegari R. Tax F. E. (2007). RPK1 and TOAD2 are two receptor-like kinases redundantly required for *Arabidopsis* embryonic pattern formation. Dev. Cell. 12, 943–956. doi: 10.1016/j.devcel.2007.04.003 17543866

[B45] Oka K. Kobayashi M. Mitsuhara I. Seo S. (2013). Jasmonic acid negatively regulates resistance to tobacco mosaic virus in tobacco. Plant Cell Physiol. 54, 1999–2010. doi: 10.1093/pcp/pct137 24071744

[B46] Osakabe Y. Maruyama K. Seki M. Satou M. Shinozaki K. Yamaguchi-Shinozaki K. (2005). Leucine-rich repeat receptor-like kinase1 is a key membrane-bound regulator of abscisic acid early signaling in *Arabidopsis* . Plant Cell. 17, 1105–1119. doi: 10.1105/tpc.104.027474 15772289 PMC1087989

[B47] Petersen T. N. Brunak S. Von Heijne G. Nielsen H. (2011). SignalP 4.0: discriminating signal peptides from transmembrane regions. Nat. Methods 8, 785–786. doi: 10.1038/nmeth.1701 21959131

[B48] Postel S. Küfner I. Beuter C. Mazzotta S. Schwedt A. Borlotti A.. (2010). The multifunctional leucine-rich repeat receptor kinase BAK1 is implicated in *Arabidopsis* development and immunity. Eur. J. Cell Biol. 89, 169–174. doi: 10.1016/j.ejcb.2009.11.001 20018402

[B49] Price M. N. Dehal P. S. Arkin A. P. (2010). FastTree 2-approximately maximum-likelihood trees for large alignments. PloS One 5, e9490. doi: 10.1371/journal.pone.0009490 20224823 PMC2835736

[B50] Roux M. Schwessinger B. Albrecht C. Chinchilla D. Jones A. Holton N.. (2011). The *Arabidopsis* leucine-rich repeat receptor-like kinases BAK1/SERK3 and BKK1/SERK4 are required for innate immunity to hemibiotrophic and biotrophic pathogens. Plant Cell. 23, 2440–2455. doi: 10.1105/tpc.111.084301 21693696 PMC3160018

[B51] Schoonbeek H.-J. Wang H.-H. Stefanato F. L. Craze M. Bowden S. Wallington E.. (2015). *Arabidopsis* EF-tu receptor enhances bacterial disease resistance in transgenic wheat. New Phytol. 206, 606–613. doi: 10.1111/nph.13356 25760815

[B52] Shiu S. H. Bleecker A. B. (2001a). Plant receptor-like kinase gene family: Diversity, function, and signaling. Sci. STKE. 2001, re22. doi: 10.1126/stke.2001.113.re22 11752632

[B53] Shiu S. H. Bleecker A. B. (2001b). Receptor-like kinases from arabidopsis form a monophyletic gene family related to animal receptor kinases. Proc. Natl. Acad. Sci. U. S. A. 98, 10763–10768. doi: 10.1073/pnas.181141598 11526204 PMC58549

[B54] Shpak E. D. Berthiaume C. T. Hill E. J. Torii K. U. (2004). Synergistic interaction of three ERECTA-family receptor-like kinases controls *Arabidopsis* organ growth and flower development by promoting cell proliferation. Development 131, 1491–1501. doi: 10.1242/dev.01028 14985254

[B55] Sun J. Li L. Wang P. Zhang S. Wu J. (2017). Genome-wide characterization, evolution, and expression analysis of the leucine-rich repeat receptor-like protein kinase (LRR-RLK) gene family in rosaceae genomes. BMC Genomics 18, 763. doi: 10.1186/s12864-017-4155-y 29017442 PMC5635495

[B56] Sun R. Wang S. Ma D. Liu C. (2018). Genome-wide analysis of LRR-RLK gene family in four *Gossypium* species and expression analysis during cotton development and stress responses. Genes (Basel) 9, 592. doi: 10.3390/genes9120592 30501109 PMC6316826

[B57] Tamura K. Stecher G. Peterson D. Filipski A. Kumar S. (2013). MEGA6: Molecular evolutionary genetics analysis version 6.0. Mol. Biol. Evol. 30, 2725–2729. doi: 10.1093/molbev/mst197 24132122 PMC3840312

[B58] Walker J. C. Zhang R. (1990). Relationship of a putative receptor protein kinase from maize to the s-locus glycoproteins of brassica. Nature 345, 743–746. doi: 10.1038/345743a0 2163028

[B59] Wang J. Wang J. Shang H. Chen X. Xu X. Hu X. (2019). *TaXa21*, a leucine-rich repeat receptor-like kinase gene associated with *TaWRKY76* and *TaWRKY62*, plays positive roles in wheat high-temperature seedling plant resistance to *Puccinia striiformis f.* sp. *tritici* . Mol. Plant Microbe Interact. 32, 32. doi: 10.1094/MPMI-05-19-0137-R 31237476

[B60] Waterhouse A. Bertoni M. Bienert S. Studer G. Tauriello G. Gumienny R.. (2018). SWISS-MODEL: homology modelling of protein structures and complexes. Nucleic Acids Res. 46, W296–W303. doi: 10.1093/nar/gky427 29788355 PMC6030848

[B61] Wei Z. Wang J. Yang S. Song Y. (2015). Identification and expression analysis of the LRR-RLK gene family in tomato (*Solanum lycopersicum*) Heinz 1706. Genome 58, 121–134. doi: 10.1139/gen-2015-0035 26207619

[B62] Wu C. Washida H. Onodera Y. Harada K. Takaiwa F. (2000). Quantitative nature of the prolamin-box, ACGT and AACA motifs in a rice glutelin gene promoter: minimal cis-element requirements for endosperm-specific gene expression. Plant J. 23, 415–421. doi: 10.1046/j.1365-313x.2000.00797.x 10929134

[B63] Wu Y. Xun Q. Guo Y. Zhang J. Cheng K. Shi T.. (2016). Genome-wide expression pattern analyses of the *Arabidopsis* leucine-rich repeat receptor-like kinases. Mol. Plant 9, 289–300. doi: 10.1016/j.molp.2015.12.011 26712505

[B64] Xu S. L. Rahman A. Baskin T. I. Kieber J. J. (2008). Two leucine-rich repeat receptor kinases mediate signaling, linking cell wall biosynthesis and ACC synthase in arabidopsis. Plant Cell. 20, 3065–3079. doi: 10.1105/tpc.108.063354 19017745 PMC2613664

[B65] Yamaguchi Y. Huffaker A. Bryan A. C. Tax F. E. Ryan C. A. (2010). PEPR2 is a second receptor for the Pep1 and Pep2 peptides and contributes to defense responses in *Arabidopsis* . Plant Cell. 22, 508–522. doi: 10.1105/tpc.109.068874 20179141 PMC2845411

[B66] Yamaguchi Y. Pearce G. Ryan C. A. (2006). The cell surface leucine-rich repeat receptor for AtPep1, an endoaenous peptide elicitor in *Arabidopsis*, is functional in transgenic tobacco cells. Proc. Natl. Acad. Sci. U. S. A. 103, 10104–10109. doi: 10.1073/pnas.0603729103 16785433 PMC1502513

[B67] Yang J. Zhang T. Li J. Wu N. Wu G. Yang J.. (2020). Chinese Wheat mosaic virus-derived vsiRNA-20 can regulate virus infection in wheat through inhibition of vacuolar- (H+)-PPase induced cell death. New Phytol. 226, 205–220. doi: 10.1111/nph.16358 31815302 PMC7065157

[B68] Yang J. Zhang F. Xie L. Song X.-J. Li J. Chen J.-P.. (2016). Functional identification of two minor capsid proteins from Chinese wheat mosaic virus using its infectious full-length cDNA clones. J. Gen. Virol. 97, 2441–2450. doi: 10.1099/jgv.0.000532 27357465

[B69] Yu X. Han J. Wang E. Xiao J. Hu R. Yang G.. (2019). Genome-wide identification and homoeologous expression analysis of PP2C genes in wheat (*Triticum aestivum l.*). Front. Genet. 10, 561. doi: 10.3389/fgene.2019.00561 31249596 PMC6582248

[B70] Zan Y. Ji Y. Zhang Y. Yang S. Song Y. Wang J. (2013). Genome-wide identification, characterization and expression analysis of *populus* leucine-rich repeat receptor-like protein kinase genes. BMC Genomics 14, 318. doi: 10.1186/1471-2164-14-318 23663326 PMC3682895

[B71] Zhang J. Z. (2003). Evolution by gene duplication: an update. Trends Ecol. Evol. 18, 292–298. doi: 10.1016/S0169-5347(03)00033-8

[B72] Zhang T. Liu P. Zhong K. Zhang F. Xu M. He L.. (2019). Wheat yellow mosaic virus NIb interacting with host light induced protein (LIP) facilitates its infection through perturbing the abscisic acid pathway in wheat. Biol. (Basel) 8, 80. doi: 10.3390/biology8040080 PMC6955802 31652738

[B73] Zhou F. Guo Y. Qiu L. J. (2016). Genome-wide identification and evolutionary analysis of leucine-rich repeat receptor-like protein kinase genes in soybean. BMC Plant Biol. 16, 58. doi: 10.1186/s12870-016-0744-1 26935840 PMC4776374

[B74] Zipfel C. Kunze G. Chinchilla D. Caniard A. Jones J. D. G. Boller T.. (2006). Perception of the bacterial PAMP EF-tu by the receptor EFR restricts *Agrobacterium*-mediated transformation. Cell 125, 749–760. doi: 10.1016/j.cell.2006.03.037 16713565

