# Supplementary figures and images for: Genome-wide identification and analysis of wheat LRR-RLK family genes following Chinese wheat mosaic virus infection
Source: Front Plant Sci. 2023 Jan 17;13:1109845. doi: 10.3389/fpls.2022.1109845 (PMC9887201; doi:10.3389/fpls.2022.1109845)

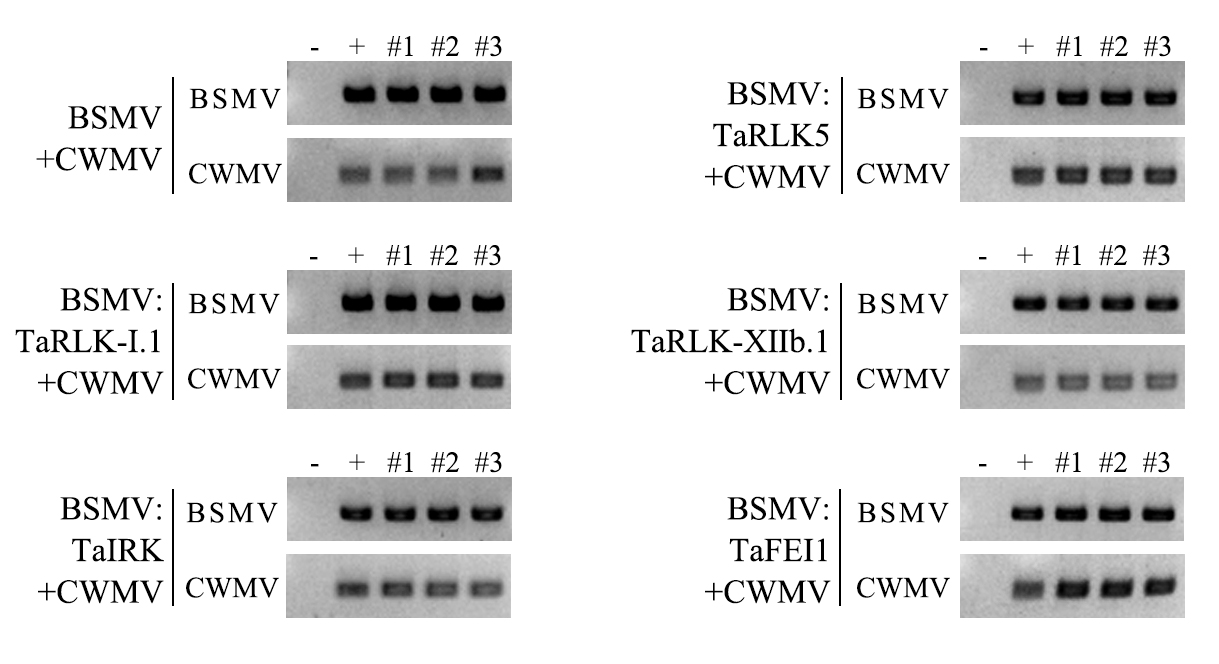

Supplement: Supplementary Figure 1 — Detection of CWMV and BSMV by RT-PCR in viuses infected leaves. The infectious clones and wheat leaves without viral infection was used as positive control (+) and negative control (-), respectively. [file Image_1.jpeg]
